# Metabolomics to identify fingerprints of carotid atherosclerosis in nonobese metabolic dysfunction-associated fatty liver disease

**DOI:** 10.1186/s12967-022-03760-6

**Published:** 2023-01-09

**Authors:** Congxiang Shao, Lishu Xu, Pingguang Lei, Wei Wang, Shiting Feng, Junzhao Ye, Bihui Zhong

**Affiliations:** 1 Department of Gastroenterology, The First Affiliated Hospital, Sun Yat-sen University, No. 58 Zhongshan II Road, Yuexiu District, Guangzhou, 510080 China; 2 Department of Gastroenterology and Hepatology, Guangdong Provincial People’s Hospital, Guangdong Academy of Medical Sciences, and Guangdong Provincial Geriatrics Institute, No. 106 Zhongshan II Road, Yuexiu District, Guangzhou, China; 3 Department of Gastroenterology, Shenzhen Baoan District Songgang People’s Hospital, No. 2, Shajiang Road, Songgang Street, Bao’an District, Shenzhen, China; 4 Department of Medical Ultrasonics, The First Affiliated Hospital, Sun Yat-Sen University, No. 58 Zhongshan II Road, Yuexiu District, Guangzhou, China; 5 Department of Radiology, The First Affiliated Hospital, Sun Yat-Sen University, No. 58 Zhongshan II Road, Yuexiu District, Guangzhou, China

**Keywords:** Cardiovascular disease, Carotid atherosclerosis, Metabolomics, Metabolic dysfunction-associated fatty liver disease, Nonobese

## Abstract

**Background/aims:**

Nonobese metabolic dysfunction-associated fatty liver disease (MAFLD) is paradoxically associated with improved metabolic and pathological features at diagnosis but similar cardiovascular diseases (CVD) prognosis to obese MAFLD. We aimed to utilize the metabolomics to identify the potential metabolite profiles accounting for this phenomenon.

**Methods:**

This prospective multicenter cross-sectional study was conducted in China enrolling derivation and validation cohorts. Liquid chromatography coupled with mass spectrometry and gas chromatography-mass spectrometry were applied to perform a metabolomics measurement.

**Results:**

The study involved 120 MAFLD patients and 60 non-MAFLD controls in the derivation cohort. Controls were divided into two groups according to the presence of carotid atherosclerosis (CAS). The MAFLD group was further divided into nonobese MAFLD with/without CAS groups and obese MAFLD with/without CAS groups. Fifty-six metabolites were statistically significant for discriminating the six groups. Among the top 10 metabolites related to CAS in nonobese MAFLD, only phosphatidylethanolamine (PE 20:2/16:0), phosphatidylglycerol (PG 18:0/20:4) and de novo lipogenesis (16:0/18:2n-6) achieved significant areas under the ROC curve (AUCs, 0.67, *p* = 0.03; 0.79, *p* = 0.02; 0.63, *p* = 0.03, respectively). The combination of these three metabolites and liver stiffness achieved a significantly higher AUC (0.92, *p* < 0.01). In obese MAFLD patients, cystine was found to be significant with an AUC of 0.69 (*p* = 0.015), followed by sphingomyelin (SM 16:1/18:1) (0.71, *p* = 0.004) and de novo lipogenesis (16:0/18:2n-6) (0.73, *p* = 0.004). The combination of these three metabolites, liver fat content and age attained a significantly higher AUC of 0.91 (*p* < 0.001). The AUCs of these metabolites remained highly significant in the independent validation cohorts involving 200 MAFLD patients and 90 controls.

**Conclusions:**

Diagnostic models combining different metabolites according to BMI categories could raise the accuracy of identifying subclinical CAS.

*Trial registration* The study protocol was approved by the local ethics committee and all the participants have provided written informed consent (Approval number: [2014] No. 112, registered at the Chinese Clinical Trial Registry, ChiCTR-ChiCTR2000034197)

**Graphical Abstract:**

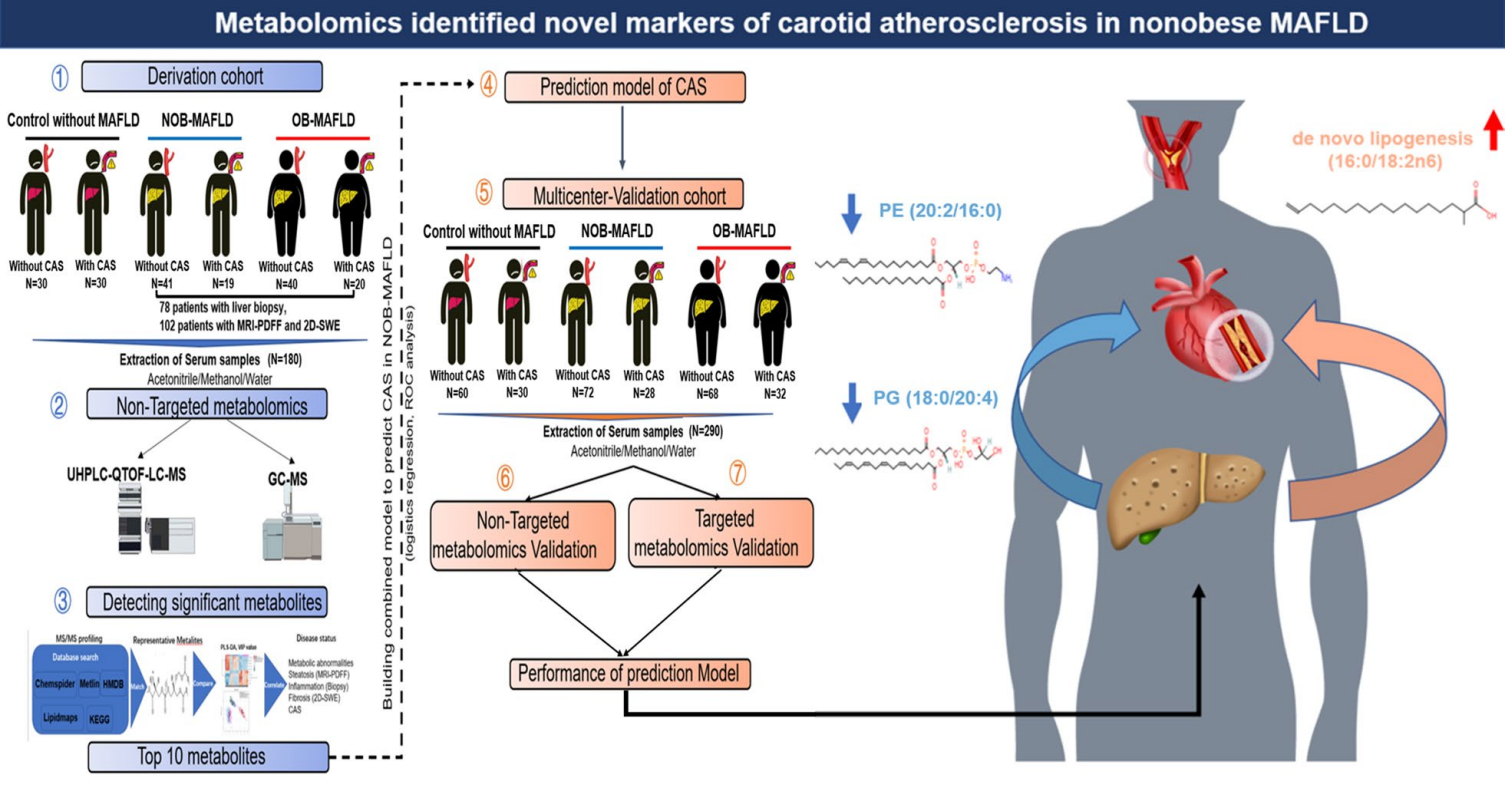

**Supplementary Information:**

The online version contains supplementary material available at 10.1186/s12967-022-03760-6.

## Background

Metabolic dysfunction-associated fatty liver disease (MAFLD) continues to be regarded as the leading cause of chronic liver disease, with an estimated prevalence of 29.8% worldwide and 29.2% in China [1, 2]. The rapid increase in MAFLD prevalence poses tremendous independent increases in the risk of cardiovascular disease (CVD), which is the top cause of mortality in MAFLD patients, with an incidence rate of 4.8 per 1000-person years [3, 4]. From a Korean nationwide health screening database involving 9,584,399 participants with a median of 10.1 follow-up years, multivariable-adjusted hazard ratios (95% confidence interval) for CVD events were 1.43 (1.41-1.45) in the MAFLD group using healthy control as reference [5]. Furthermore, a recent meta-analysis, consisting of 16 observational prospective and retrospective studies, reported a 64% increased risk in both fatal and nonfatal cardiovascular events over 7 years in patients with MAFLD [4]. Increased carotid intimal-medial thickness (CIMT) and carotid plaques measured by carotid ultrasound were used to detect subclinical carotid atherosclerosis and evaluate the future risk of CVD [6]. However, it is not practical to use these factors for patients worldwide due to the high prevalence of MAFLD. Therefore, establishing a precise strategy for screening carotid atherosclerosis (CAS) is urgently needed to prevent the development of CVD in MAFLD.

Although the links between MAFLD and CVD/CAS have been mostly attributed to their shared risk factors, such as obesity [7], it has been reported that the prevalence and accumulated incident percentages of CVD among lean MAFLD patients [body mass indexes (BMI) < 23 kg/m^2^] were not lower than those among overweight patients in both cross-sectional and long-term longitudinal studies [8]. More importantly, two meta-analyses demonstrated that nonobese patients presented similar metabolic characteristics to obese individuals but with lower levels of anthropometric and routine metabolic indices, including BMI, waist circumstance (WC), triglycerides (TG), total cholesterol (CHOL), fasting blood glucose (FBG), alanine aminotransferase (ALT), which suggests that the value of screening for CAS by using these traditional metabolic markers is limited [8–10].

Metabolic dysregulation plays a critical role in different stages of MAFLD, which is also highly associated with CVD development [11]. Serum metabolomics analysis as a high-throughput method, provides data on the detailed metabolic changes in MAFLD and emerging studies have identified specific metabolites involved in steatosis, inflammation and fibrosis progression including free fatty acids, bile acids, saccharides, amino acids, and phospholipids, making this method promising in identifying biomarkers for assessing MAFLD [12, 13]. Indeed, some of these metabolic markers were also been shown to have additional prognostic value for CVD according to a recently published meta-analysis including 19 studies (45,420 subjects, 5954 events) [14]. Thus, the changes in metabolites derived from omics approaches are likely to reflect specific pathways of CAS and MAFLD, making them effective candidates for distinguishing the CAS in the nonobese individuals with MAFLD [10].

In the present study, both ultrahigh-performance liquid chromatography coupled with quadrupole time-of-flight mass spectrometry (UHPLC-QTOF-MS) and gas chromatography-mass spectrometry (GC-MS) were utilized to identify the different metabolite profiles between nonobese and obese MAFLD patients with and without CAS. We aimed to investigate whether a combination of serum metabolites could accurately predict the presence of CAS with further prospective independent validation.

## Methods

This cross-sectional study was conducted at three MAFLD centers in Southern China including the first affiliated hospital, Sun Yat-sen University, Guangdong provincial people’s hospital and Shenzhen Songgang District People's Hospital. The study protocol was approved by the local ethics committee and all the participants have provided written informed consent (Approval number: [2014] No. 112, registered at the Chinese Clinical Trial Registry, ChiCTR-ChiCTR2000034197). The derivation cohort of this study involved 120 MAFLD patients and 60 controls consecutively recruited from the first affiliated hospital, Sun Yat-sen University between October 2015 and May 2017. The diagnosis of MAFLD is based on the detection of liver steatosis together with the presence of at least one of three criteria that include overweight or obesity, type 2 diabetes mellitus (T2DM) or clinical evidence of metabolic dysfunction [15, 16]. For the derivation study, liver steatosis was defined as the liver fat content (LFC) of more than 5% estimated via magnetic resonance imaging-based proton density fat fraction (MRI-PDFF) [17]. The prospective independent validation cohort consecutively included 200 MAFLD patients and 90 controls from the first affiliated hospital, Sun Yat-sen University, Guangdong provincial people’s hospital and Shenzhen Songgang District People's Hospital from June 2017 to December 2021. For the validation cohort, liver steatosis was detected via FibroScan in Guangdong provincial people’s hospital and Shenzhen Songgang District People's Hospital. Fatty liver was defined as the controlled attenuation parameter (CAP) with FibroScan of more than 254 dB/m [18].

All the participants were at least 18 years old with complete anthropometric measurements as well as the laboratory and imaging examination results. Participants were excluded if they met any of the followings: habitual alcohol consumption or significant alcohol intake (≥ 70 g/week in women and ≥ 140 g/week in men); positive hepatitis B surface antigen or positive antibody against hepatitis C virus; autoimmune liver disease; endocrine disorders (e.g., hypothyroidism); competing etiologies of liver disease inducing hepatic steatosis (e.g., consumption of tamoxifen and amiodarone); being a trained athlete with a hypertrophic muscle mass; malignancies; pregnancy.

### Clinical assessment

Patients were required to fill in a structured questionnaire involving the alcohol intake, smoking and past medical history. A standard physical examination was conducted by specialized doctors to obtain the anthropometric data, including weight, height, waist circumference (WC) and hip circumference, and blood pressure. Based on the recommendation of WHO expert consultation, the MAFLD patients with a BMI lower than 25 kg/m^2^ were regarded as nonobese MAFLD. The increased WC was defined by the cutoff value of 90 cm and 80 cm in men and women, respectively [18, 19].

After an overnight fast, the venous blood was drawn. Blood was centrifuged and plasma was analyzed for liver biochemistry, lipids, biochemistry and insulin. The homeostatic model assessment of insulin resistance (HOMA-IR), calculated as fasting blood glucose (FBG, mmol/L) × fasting insulin (FINS, μU/mL)/22.5, was utilized to evaluate the insulin resistance (IR). The cutoff value of 2.5 was defined as IR [15].

### Quantification of liver fat content via MRI-PDFF

During the initial clinical assessment, MRI-PDFF was conducted via 3.0-T scanner (Siemens 3.0T MAGNETOM Verio). The sequence parameter setting and operation procedure were in accordance with that reported in our previous studies [20–22]. In brief, TE1 2.5 ms, TE2 3.7 ms, 5.47 ms for repetition; 5 flip angles; ± 504.0 kHz per pixel receiver bandwidth and a slice thickness of 3.0 mm. After the images of fat-water separation were attained, data concerning LFC, pancreas fat content (PFC) and abdominal subcutaneous fat thickness (ASFT) were estimated. In addition, consistent with the previous clinical trials, we classified fatty liver into mild, moderate and severe with the LFC of 5.36-15.36%, 15.36-20.35% and > 20.35%, respectively [23].

### Measurement of CAP and liver stiffness

CAP and liver stiffness measurements were conducted via transient elastography (FibroScan‐402, Echosens, France) with either an M- or an XL-probe along with the instructions. Patients were placed in a supine position with the right arm elevated above the head and extended to the maximum. A success rate of > 60% and ≥ 10 eligible acquisitions were adopted as valid measurement results. Based on our previous study, the cutoff value of 6.1 kPa was applied to define the presence of fibrosis. A cutoff value of 254 dB/m was utilized to define fatty liver [17]. A preliminary study for 50 NAFLD patients in three centers showed that the kappa statistic of interobserver and interobserver reliability for CAP were 0.89 and 0.91, respectively, and 0.88 and 0.87 for liver stiffness measurement.

### Histological assessment

Seventy-eight patients underwent liver biopsy to confirm the diagnosis of fatty liver. The details of liver biopsy and histological assessment were listed in the Additional file 1.

### Evaluation of carotid atherosclerosis

Two specialized sonographers, with more than 10 years of experience, performed the high-resolution B-mode ultrasonography examination to measure the CIMT and detect the presence of CAS. Three measurements were performed to gain the average of CIMT for further analysis. CIMT > 1.0 mm was regarded as carotid intima-media thickening. Besides, a plaque was defined as focal thickening of intima-media > 0.5 mm or 50% of surrounding intima-media into the arterial lumen, or a focal thickening > 1.5 mm [24, 25]. We conducted a preliminary study involving 100 NAFLD patients recruited from our center. The results showed that the kappa statistic of interobserver and interobserver reliability for carotid intima-media thickness and carotid plaque between the two sonographers were 0.92 and 0.96, respectively.

### Metabolomics

Samples preparations and detailed parameter settings were shown in the Additional file 1. For UHPLC-QTOF-MS, liquid chromatographic analysis was performed on a Waters ACQUITY UPLC Iclass system (Waters Ltd. USA) coupled with an ACQUITY UPLC BEH C18 column. The original data was imported into the Progenesis QI (Nonlinear Dynamics Waters, UK) software to extract the matched peak of filtering noise peak and EZinfo 3.0 for Waters (Umetrics, Sweden) software was utilized for statistical analysis.

Phospholipid compound identification and analysis based on the phospholipid cleavage law and online databases METLIN metabolite MSMS database and LipidMaps. The identification results were further confirmed by accurate mass number and secondary ion fragments collected by high resolution mass spectrometry.

For GC-MS, An Agilent 7890 GC system equipped with a 5977-quadrupole mass selective detector (MSD, Agilent, Santa Clara, CA) and a HP-5MS column (40 m × 0.25 mm inner diameter × 0.25 μm film thickness, Agilent, Santa Clara, CA) was employed to acquire metabolic profiles of the derivatized products. The mass spectrum was acquired in full-scan mode from 50 to 550 m/z. Recorded mass spectra were compared with those stored in the National Institute of Standards and Technology (NIST) US Government library. Quantitative analysis was performed by measuring total ion current chromatographic peak areas. Detailed parameter settings of targeted metabonomics were shown in the Additional file 1. All the metabonomics analysis were conducted in instrumental analysis and research center of Sun Yat-sen University.

### Statistical analysis

Statistical analysis was performed via SPSS 25.0 software (IBM, Chicago, IL, USA) and R language version 3.3.3. Normally distributed data were expressed as the mean ± standard deviation (SD) while the non-normally distributed variables were expressed as the median with interquartile ranges (IQR). The Kruskal-Wallis test was used to analyse continuous variables and categorical variables were compared via Chi-squared test. Partial least squares-discriminant analysis (PLS-DA) was performed on samples to determine the overall metabolic differences. LASSO Cox regression and backward stepwise logistic regressions were utilized to explore the factors that were associated with CAS among nonobese patients and obese patients in the derivation cohort and validation cohort. The receiver operating characteristic (ROC) curve analysis was performed for the predictive factors of CAS. KEGG pathway enrichment analysis revealed the main differential metabolic pathways. Pearson correlation analysis was conducted to test the correlation between different metabolites and metabolic indexes and histological indexes, respectively. A two-sided *P* value of < 0.05 was considered statistically significant.

## Results

### Clinical characteristics of the derivation cohort

**Fig. 1:**
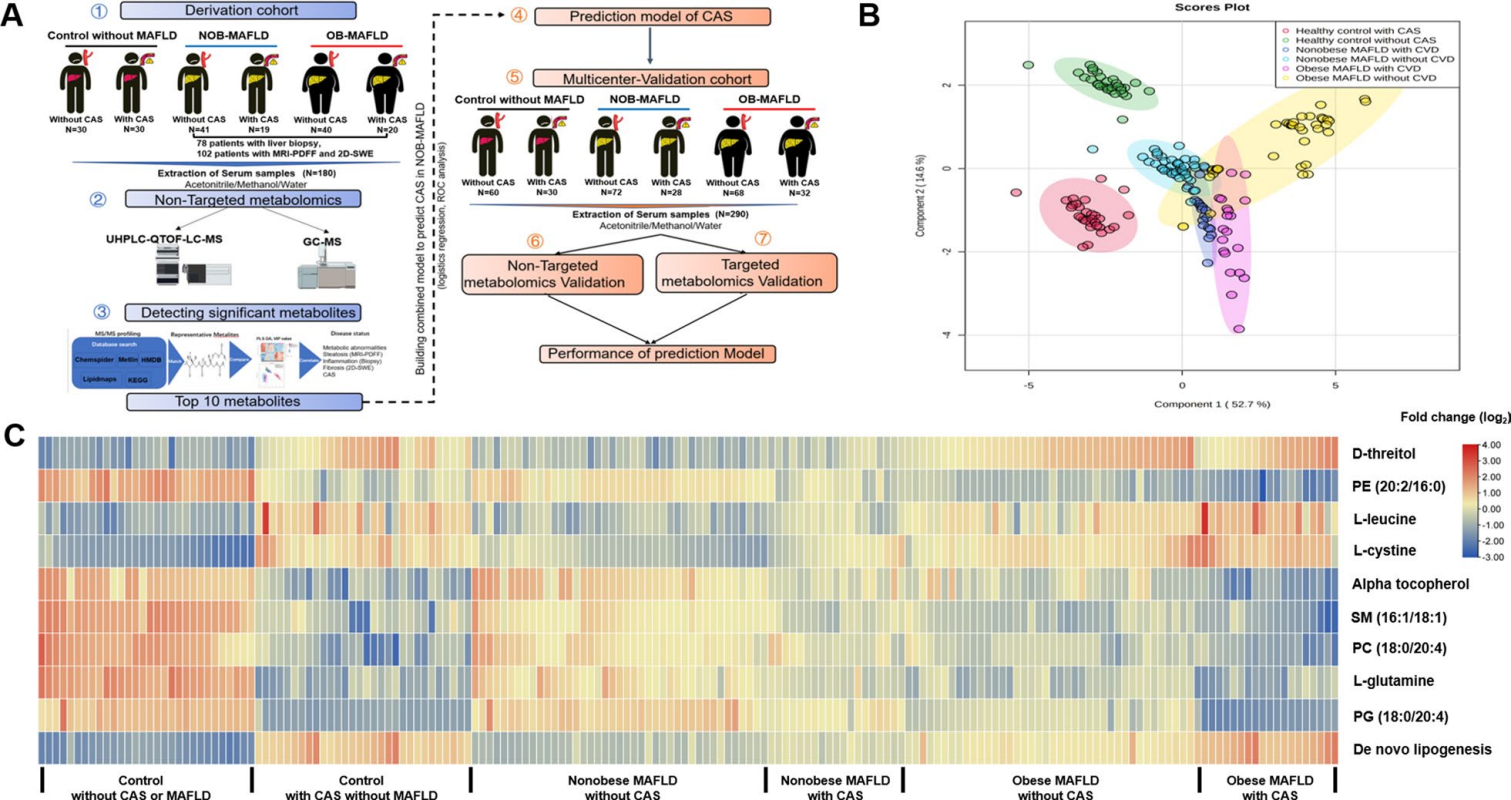
**A** The flow diagram of the present study. **B** The serum partial least squares-discriminant analysis (PLS-DA) score plot among non-MAFLD with or without CAS, nonobese MAFLD with or without CAS, and obese MAFLD with or without CAS. **C** The heat map of all differential metabolites, and metabolites with top 10 VIP value based on the partial least squares-discriminant analysis (PLS-DA), respectively. NOB-MAFLD, nonobese metabolic associated fatty liver disease; OB-MAFLD, obese metabolic associated fatty liver disease; CAS, carotid atherosclerosis; UHPLC-QTOF-LC-MS, ultrahigh-performance liquid chromatography coupled with quadrupole time-of-flight mass spectrometry; QC-MS, gas chromatography-mass spectrometry

As presented in Fig. 1A, a total of 180 individuals were recruited in the derivation cohort, which consisted of 120 (66.7%) MAFLD patients and 60 (33.3%) controls. Controls were classified into two groups based on the presence of CAS. The MAFLD patients were further categorized into groups of nonobese MAFLD patients with/without CAS and obese MAFLD patients with/without CAS. The clinical characteristics, including anthropometrical data, metabolomics and imaging examination results are displayed in Table 1. No significant differences were found in age, gender or blood pressure among the six groups. Regarding hepatic markers and metabolic characteristics, alanine transaminase (ALT), high-density lipoprotein-cholesterol (HDL-C), fasting insulin (FINS) and uric acid (UA) showed significant differences, while there was no significant difference among the groups in aspartate transaminase (AST), total cholesterol (CHOL), triglycerides (TG), low-density lipoprotein-cholesterol (LDL-C), fasting blood glucose (FBG) and homeostatic model assessment of insulin resistance (HOMA-IR). In addition, concerning the imaging examination results, significant differences were found in LFC, PFC and abdominal subcutaneous fat thickness (ASFT) among the six groups. However, the groups did not differ in liver stiffness.

**Table 1:** Clinical characteristics of deviation cohort

Characteristics	Non-MAFLD without CAS (n = 30)	Nonobese MAFLD		Obese MAFLD		Non-MAFLD with CAS (n = 30)	*P*
		Without CAS (n = 41)	With CAS (n = 19)	Without CAS (n = 40)	With CAS (n = 20)		
Age (year)	44.6 ± 11.9	43.6 ± 11.7	42.4 ± 11.7	45.2 ± 10.8	44.1 ± 12.1	44.5 ± 9.9	0.82
Male, n (%)	18 (60.0)	28 (66.9)	13 (68.4)	26 (65.0)	14 (70.0)	19 (63.3)	0.53
Body mass index (kg/m^2^)	23.6 ± 3.8 bcdef	23.1 ± 2.2 adef	23.1 ± 1.7 adef	28.4 ± 2.6 abce	31.1 ± 5.1 abcdf	27.5 ± 2.4 abce	< 0.001
Waist circumstance (cm)	81.7 ± 7.4 def	82.2 ± .6.4 def	81.7 ± 6.2 def	93.8 ± 6.9 abc	95.1 ± 6.3 abc	91.5 ± 6.0 abc	< 0.001
Waist-hip-ratio	0.87 ± 0.05 def	0.87 ± 0.05 def	0.87 ± 0.05 def	0.91 ± 0.05 abc	0.94 ± 0.05 abc	0.90 ± 0.06 abc	< 0.001
SBP (mmHg)	127 ± 13	129 ± 12	132 ± 23	130 ± 17	133 ± 15	128 ± 12	0.71
DBP (mmHg)	86 ± 10	86 ± 11	85 ± 15	89 ± 13	91 ± 14	85 ± 11	0.44
ALT (U/L)^a^	28 (18–34) bd	40 (21–81) a	30 (20–52)	42 (22–97) a	36 (31–73)	34 (24–54)	0.031
AST (U/L)^a^	25 (20–28)	30 (20–40)	28 (23–52)	31 (20–97)	31 (25–50)	29 (25–75)	0.26
Total cholesterol (mmol/L)	5.1 ± 1.0	5.4 ± 1.1	5.0 ± 0.9	5.2 ± 0.9	5.3 ± 0.8	5.1 ± 0.6	0.55
Triglycerides (mmol/L)	1.6 ± 0.8	2.1 ± 0.7	2.1 ± 0.5	2.3 ± 0.9	2.4 ± 0.7	2.1 ± 0.5	0.23
HDL-cholesterol (mmol/L)	1.2 ± 0.2 df	1.3 ± 0.4 def	1.2 ± 0.3	1.1 ± 0.7 ab	1.0 ± 0.5 b	1.1 ± 0.5 ab	0.017
LDL-cholesterol (mmol/L)	3.1 ± 0.8	3.3 ± 0.8	3.4 ± 0.7	3.6 ± 0.9	3.7 ± 0.5	3.6 ± 0.8	0.16
FBG (mmol/L)	4.9 ± 0.7	5.0 ± 1.4	5.3 ± 1.1	5.4 ± 0.9	5.4 ± 0.4	5.2 ± 0.8	0.22
Fasting insulin (μU/mL)	9.0 ± 2.9 e	8.8 ± 2.9 e	9.0 ± 1.3 e	9.4 ± 2.8 e	19.3 ± 3.8 abcdf	9.1 ± 2.1 e	0.001
HOMA-IR^a^	1.54 (0.90–2.36)	1.61 (1.18–2.23)	1.77 (1.38–2.67)	1.60 (0.96–2.40)	2.69 (1.43–6.76)	1.70 (1.25–2.56)	0.08
Uric acid (μmol/L)	372 ± 92 def	400 ± 115 def	394 ± 79 def	458 ± 97 abc	475 ± 133 abc	432 ± 101 abc	0.001
Liver fat content (%)^a^	3.8 (3.1–4.3) bcde	10.8 (6.7–17.1) adef	9.3 (6.8–17.4) adef	15.1 (10.5–23.3) abcf	16.0 (11.1–24.6) abcf	3.6 (3.0–4.5) bcde	0.001
Pancreas fat content (%)^a^	1.7 (1.2–2.3) cde	1.8 (1.3–2.5) de	1.9 (1.1–2.9) adef	2.6 (1.8–4.3) abcf	3.6 (1.5–6.9) abcf	1.6 (1.1–2.2) cde	0.002
ASFT (mm)	22.8 ± 8.2 bf	19.3 ± 6.8 ade	20.1 ± 6.9 e	23.5 ± 6.2 bf	26.0 ± 6.5 bcf	20.4 ± 7.0 ade	0.01
Liver stiffness (kPa)	5.6 ± 2.3	5.8 ± 1.3	6.5 ± 2.2	6.1 ± 1.0	7.7 ± 2.5	5.8 ± 1.8	0.08

a, b, c, d, e, f-refer to statistic significant after post-hoc multiple comparisons with Bonferroni adjustments when compared with Non-MAFLD without CAS(a), Nonobese MAFLD without (b) or with (c) carotid atherosclerosis, obese MAFLD without (d) or with (e) carotid atherosclerosis, and non-MAFLD with CAS (f)

MAFLD, metabolic dysfunction-associated fatty liver disease; CAS, carotid atherosclerosis; SBP, systolic blood pressure; DBP, diastolic blood pressure; ALT, alanine aminotransferase, AST, aspartate aminotransferase; HDL, high-density lipoprotein-cholesterol; LDL, low-density lipoprotein-cholesterol; FBG, fasting blood glucose; HOMA-IR, homeostasis model assessment of insulin resistance; ASFT, abdominal subcutaneous fat thickness

^a^Continuous variables are expressed as median with IQR for non-Gaussian distribution

### Metabolic characteristics of the derivation cohort

PLSDA was performed on samples to determine the overall metabolic differences between the six groups (Fig. 1B). From the UHPLC-QTOF-MS analysis, among 573 serum metabolites identified, 36 metabolites were found to significantly differentiate the groups (variable importance in the projection, VIP > 1 and *p* < 0.05). From the GC–MS analysis, among the 152 serum metabolites analyzed, 20 metabolites were statistically significant for discriminating among the six groups (VIP > 1 and *p* < 0.05). These serum metabolites were amino acids, carbohydrates, vitamins and lipids. Taken these 56 significant metabolites together, the serum metabolites with the top 10 VIP values were shown in Table 2 and Fig. 1C.

**Table 2:** Top 10 metabolites differentially expressed among five groups

Metabolites	VIP-value (PLS-DA)	*P*
Alpha tocopherol	2.68	< 0.001
PE (20:2/16:0)	2.55	< 0.001
l-glutamine	2.51	< 0.001
PC (18:2/20:2)	2.44	< 0.001
SM (16:1/18:1)	2.36	< 0.001
d-threitol	2.28	< 0.001
PG (18:0/20:4)	2.22	< 0.001
De novo lipogenesis (16:0/18:2n-6)	2.07	0.001
l-leucine	1.99	0.001
Cystine	1.95	0.002

VIP Values for the variable importance in the project

**Fig. 2:**
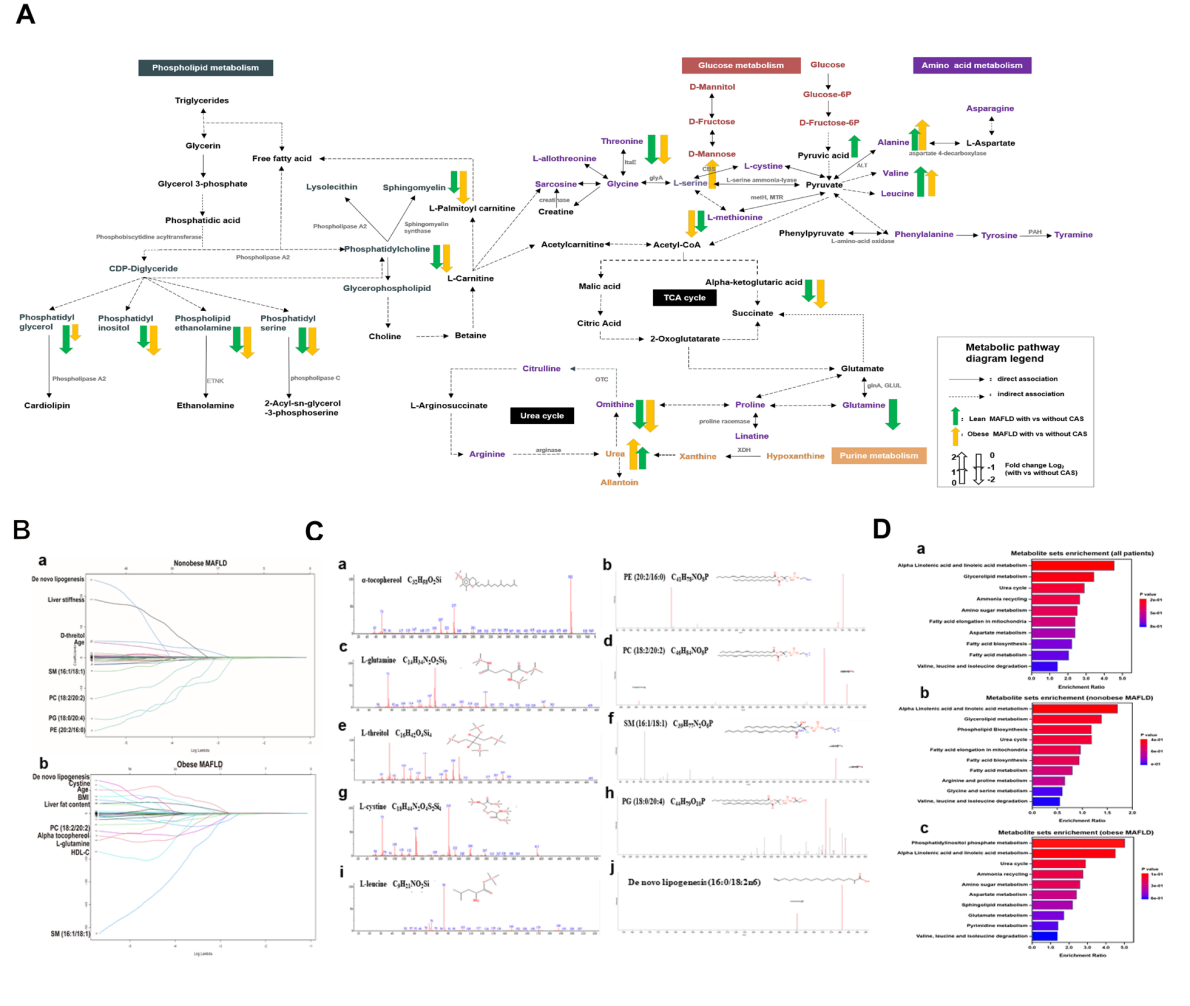
**A** Schematic scheme of disturbed metabolic pathways. Metabolites marked in green and yellow indicate metabolites concentration comparison between nonobese MAFLD with vs without CAS, and obese MAFLD with vs without CAS, respectively. ItaE, low specificity l-threonine aldolase; glyA, glycine hydroxy methyltransferase; CBS, cystathionine beta-synthase; metH, MTR, 5-methyltetrahydrofolate–homocysteine methyltransferase; ALT, alanine transaminase.PAH, proclavaminate amidinohydrolase; OTC, ornithine carbamoyltransferase; GlnA, glutamine synthetase; XDH, xanthine dehydrogenase/oxidase; ETNK, ethanolamine kinase. **B** LASSO regression analysis of factors associated with carotid atherosclerosis among nonobese MAFLD and obese MAFLD patients, respectively. Each curve corresponds to an index. **C** Mass spectrograms of metabolites with top 10 VIP value based on the partial least squares-discriminant analysis (PLS-DA). **D** The diagram of metabolites for KEGG pathway enrichment analysis among all MAFLD, nonobese MAFLD and obese MAFLD, respectively

A schematic scheme of proposed metabolic pathways was presented to visualize the interaction between the differential metabolites (Fig. 2A). The metabolites with significant changes were mapped onto several biochemical processes including phospholipid metabolism, glucose metabolism, amino acid metabolism, urea cycle and tricarboxylic acid cycle.

### Factors associated with carotid atherosclerosis in nonobese and obese MAFLD patients of deviation cohort

We performed the LASSO Cox regression model to explore the association between CAS and metabolites (Fig. 2B). The mass spectrograms of these statistically different metabolites were shown in Fig. 2C. Compared to obese MAFLD, the KEGG pathway enrichment analysis revealed that the main differential metabolic pathways were glycolipid metabolism, phospholipid biosynthesis, fatty biosynthesis and metabolism, arginine and proline metabolism, and glycine and serine metabolism (Fig. 2D).

### Metabolomics validation

**Fig. 3:**
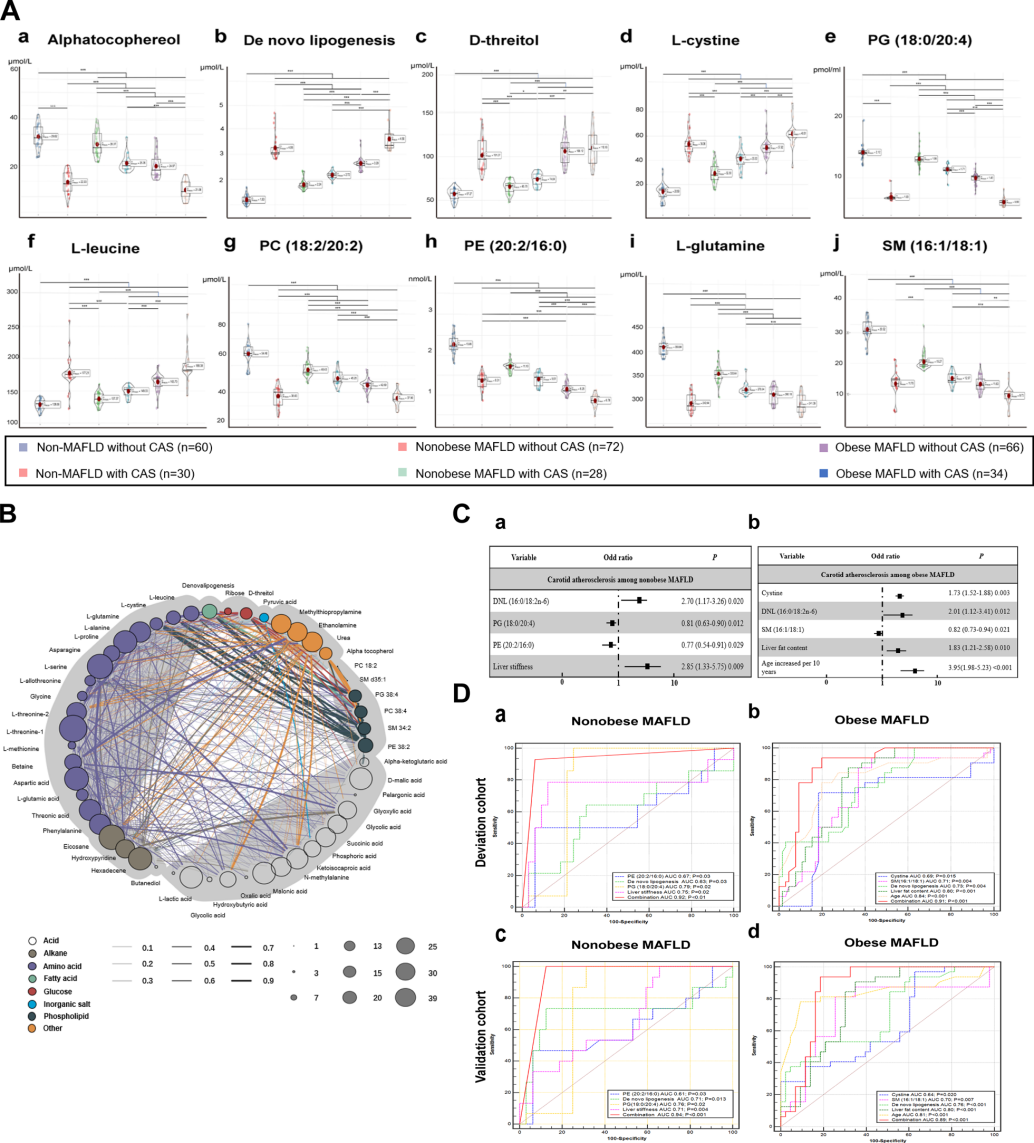
**A** The violin plot of concentration comparison of the metabolites with top 10 VIP value among healthy controls, nonobese MAFLD patients with/without CAS and obese MAFLD patients with/without CAS in the validation cohort. Group 1: non-MAFLD without CAS; Group 2. non-MAFLD with CAS; Group 3: nonobese MAFLD patients without CAS; Group 4: nonobese MAFLD patients with CAS; Group 5: obese MAFLD patients without CAS; Group 6: obese MAFLD patients with CAS. **B** Correlation network graph of differential metabolite. The correlation network was constructed according to their correlation coefficient and the number of metabolites with correlation coefficient greater than 0.5. **C** Multivariate logistic regression for serum metabolites associated with carotid atherosclerosis in the nonobese and obese MAFLD patients. **D** Receiver operator characteristic (ROC) curve of metabolites that predict carotid atherosclerosis for nonobese MAFLD (a) and obese MAFLD (b) in derivation cohort, nonobese MAFLD (c) and obese MAFLD (d) in validation cohort

We further evaluated the diagnostic performance of the significant metabolites and their combinations in an independent validation cohort involving 200 MAFLD patients and 90 non-MAFLD controls. The clinical characteristics are shown in Table 3. The comparison between six subgroups had similar trends to those in the deviation cohort. Targeted metabolomics analysis was performed for the serum metabolites with the top 10 VIP and the chromatograms of these metabolites were shown in Additional file 1: Fig. S1, respectively. All the metabolites distributed differently among the six groups (Fig. 3A). In the untargeted metabolomics, there were various correlations among main differential metabolites (Fig. 3B).

**Table 3:** Clinical characteristics of validation cohort

Characteristics	Non-MAFLD without CAS (n = 60)	Nonobese MAFLD		Obese MAFLD		Non-MAFLD with CAS (n = 30)	*P*
		Without CAS (n = 72)	With CAS (n = 28)	Without CAS (n = 68)	With CAS (n = 32)		
Age (year)	42.6 ± 10.1	44.6 ± 9.8	43.4 ± 10.3	44.2 ± 10.2	45.1 ± 9.9	44.3 ± 9.2	0.58
Male, n (%)	40 (66.7%)	42 (61.1%)	17 (60.7%)	44 (64.7%)	20 (62.5%)	18 (60.0%)	0.64
Body mass index (kg/m^2^)	22.9 ± 2.2 bcdef	23.5 ± 2.8 acdef	23.6 ± 2.7 adef	28.8 ± 3.1 abcef	32.2 ± 2.1 abcdf	31.5 ± 2.5 abcde	< 0.001
Waist circumstance (cm)	81.7 ± 7.5 def	82.2 ± .6.4 def	83.8 ± 7.2 def	92.5 ± 6.9 abc	93.8 ± 7.2 abc	92.2 ± 7.0 abc	< 0.001
Waist-hip- ratio	0.85 ± 0.04 def	0.87 ± 0.03 def	0.88 ± 0.04 def	0.92 ± 0.06 abc	0.94 ± 0.05 abc	0.92 ± 0.04 abc	< 0.001
SBP (mmHg)	125 ± 11	126 ± 13	132 ± 21	133 ± 15	135 ± 11	131 ± 12	0.68
DBP (mmHg)	85 ± 11	87 ± 9	86 ± 12	91 ± 12	92 ± 10	93 ± 8	0.47
ALT (U/L)^a^	22 (15–31) bcdef	42 (21–59) aef	45 (28–72) a	39 (22–77) aef	48 (32–83) abd	46 (31–86) abd	0.025
AST (U/L)^a^	25 (18–27)	32 (21–39)	33 (22–49)	31 (21–77)	31 (24–52)	30 (20–53)	0.31
Total cholesterol (mmol/L)	4.9 ± 0.8	5.2 ± 1.2	5.1 ± 0.9	5.3 ± 1.0	5.3 ± 0.7	5.1 ± 0.9	0.79
Triglycerides (mmol/L)	1.5 ± 0.7	2.2 ± 0.7	2.2 ± 0.8	2.4 ± 0.5	2.4 ± 0.4	2.2 ± 0.6	0.34
HDL-cholesterol (mmol/L)	1.3 ± 0.4 d	1.3 ± 0.2 def	1.2 ± 0.3 ef	1.1 ± 0.7 ab	0.9 ± 0.6 bc	1.0 ± 0.7 bc	0.008
LDL-cholesterol (mmol/L)	3.0 ± 0.7	3.2 ± 0.9	3.5 ± 0.6	3.6 ± 0.9	3.8 ± 0.4	3.6 ± 0.7	0.13
FBG (mmol/L)	4.4 ± 0.8 bcdef	5.2 ± 1.0 a	5.3 ± 0.9 a	5.4 ± 1.0 a	5.5 ± 0.8 a	5.5 ± 0.9 a	0.045
FINS (μU/mL)^a^	8.9 ± 1.5 def	9.0 ± 1.9 ef	9.3 ± 1.3 ef	9.4 ± 1.7 aef	15.4 ± 2.8 abcd	13.9 ± 1.8 abcd	0.008
HOMA-IR^a^	1.56 (1.0–2.25)	1.65 (1.28–2.11)	1.68 (1.38–2.89)	1.70 (0.96–2.40)	2.39 (1.83–5.62)	2.09 (1.13–2.62)	0.14
Uric acid (μmol/L)	368 ± 72 def	389 ± 95 def	414 ± 79 def	475 ± 87 abc	496 ± 94 abc	478 ± 77 abc	0.001
CAP (dB/m)	203 ± 14 bcde	278 ± 27 adef	285 ± 22 adef	310 ± 22 abcf	313 ± 28 abcf	215 ± 17 bcde	< 0.001
Liver stiffness (kPa)	5.9 ± 1.8 e	5.2 ± 0.8 de	5.7 ± 1.1 de	6.6 ± 1.3 bcf	8.0 ± 2.3 abcdf	5.3 ± 0.6 de	0.038

a, b, c, d, e, f, g-refer to statistic significant after post-hoc multiple comparisons with Bonferroni adjustments when compared with Non-MAFLD without CAS (a), Nonobese MAFLD without (b) or with (c) carotid atherosclerosis, obese MAFLD without (d) or with (e) carotid atherosclerosis and non-MAFLD with CAS (f).

MAFLD, metabolic dysfunction-associated fatty liver disease; CAS, carotid atherosclerosis; SBP, systolic blood pressure; DBP, diastolic blood pressure; ALT, alanine aminotransferase, AST, aspartate aminotransferase, HDL, high-density lipoprotein-cholesterol; LDL, low-density lipoprotein-cholesterol; FBG, fasting blood glucose; HOMA-IR, homeostasis model assessment of insulin resistance; CAP, controlled attenuation parameter

^a^Continuous variables are expressed as median with IQR for non-Gaussian distribution

### Factors associated with carotid atherosclerosis in the validation cohort

The univariate analysis in nonobese patients revealed that liver stiffness, PE (20:2/16:0), PG (18:0/20:4) and De novo lipogenesis (16:0/18:2n-6) were associated with CAS (Table 4). After multivariate analysis, liver stiffness (OR 2.85, 95% CI 1.33–5.75, *p* = 0.009), PE (20:2/16:0) (OR 0.77, 95%CI 0.54–0.91, *p* = 0.029), PG (18:0/20:4) (OR 0.81, 95%CI 0.63–0.90, *p* = 0.012) and de novo lipogenesis (16:0/18:2n-6) (OR 2.70, 95%CI 1.17–3.26, *p* = 0.020) remained significant factors (Fig. 3C). For obese patients, age increased per 10 years, LFC, SM (16:1/18:1), de novo lipogenesis (16:0/18:2n-6) and cystine were significantly associated with CAS. Multivariate analysis showed that age increased per 10 years (OR 3.95; 95% CI 1.98–5.23, *p* < 0.001), LFC (OR 1.83; 95% CI 1.21–2.58, *p* = 0.01), SM (16:1/18:1) (OR 0.82; 95% CI 0.73–0.94, *p* < 0.001),de novo lipogenesis (16:0/18:2n-6) (OR 2.01; 95% CI 1.12–3.41, *p* = 0.012) and cystine (OR 1.73; 95% CI 1.52–1.88, *p* = 0.003) were independent predictors of CAS (Fig. 3C).

**Table 4:** Serum metabolites associated with carotid atherosclerosis in the nonobese and obese MAFLD patients

Factors	Nonobese MAFLD		Obese MAFLD	
	Univariate		Univariate	
	OR (95%CI)	*P*	OR (95%CI)	*P*
Male	0.99 (0.31–3.20)	0.99	1.71 (0.34–8.69)	0.52
Age increased per 10 years	0.95 (0.67–1.34)	0.76	3.32 (1.57–4.85)	< 0.001
Body mass index	1.02 (0.78–1.34)	0.88	1.03 (0.95–1.11)	0.46
Increased WC	0.98 (0.90–1.08)	0.76	1.03 (0.97–1.12)	0.56
Hypertension	1.02 (0.98–1.05)	0.32	1.01 (0.98–1.04)	0.60
ALT > 40 U/L	0.98 (0.96–1.01)	0.07	0.99 (0.98–1.01)	0.51
CHOL > 5.7 mmol/L	0.89 (0.53–1.48)	0.65	1.10 (0.59–2.06)	0.77
Triglycerides > 1.7 mmol/L	2.22 (0.61–8.08)	0.23	1.56 (0.83–2.92)	0.17
LDL-C > 3.4 mmol/L	1.02 (0.50–2.09)	0.96	1.40 (0.57–3.40)	0.46
HOMA-IR > 2.5	1.12 (0.77–1.62)	0.57	0.97 (0.46–2.05)	0.95
Liver stiffness	2.59 (1.21–5.54)	0.014	1.52 (0.95–2.42)	0.08
Liver fat content	0.97 (0.90–1.05)	0.52	1.52 (1.08–2.14)	0.016
Pancreas fat content	1.27 (0.75–2.17)	0.37	1.05 (0.89–1.25)	0.57
Alpha tocophereol	1.62 (0.52–2.43)	0.31	1.35 (0.92–1.94)	0.15
PE (20:2/16:0)	0.68 (0.45–0.98)	0.038	1.26 (0.83–1.49)	0.31
l-glutamine	0.73 (0.29–1.83)	0.50	1.03 (0.98–1.06)	0.81
PC (18:2/20:2)	0.59 (0.42–0.86)	0.44	0.97 (0.89–1.05)	0.89
SM (16:1/18:1)	1.59 (0.58–2.69)	0.22	0.79 (0.58–0.96)	0.025
d-threitol	1.22 (0.42–2.49)	0.65	1.32 (0.73–2.63)	0.62
PG (18:0/20:4)	0.76 (0.58–0.92)	0.019	2.02 (0.93–2.99)	0.54
DNL (16:0/18:2n-6)	2.42 (1.08–3.40)	0.032	2.53 (1.23–3.98)	0.020
Cystine	0.68 (0.22–1.72)	0.48	1.69 (1.44–1.93)	0.008
l-leucine	1.12 (0.92–1.24)	0.53	1.55 (0.91–2.59)	0.29

ALT, alanine aminotransferase; CHOL, total cholesterol; LDL, low-density lipoprotein-cholesterol; HOMA-IR, homeostasis model assessment of insulin resistance; FBG, fasting blood glucose; PE, phosphatidylethanolamine; PC, phosphatidylcholine; SM, sphingomyelin; PG, phosphatidylglycerol; DNL, De novo lipogenesis

### Correlation between the predictive metabolites of CAS, and metabolic and pathological index

In nonobese MAFLD with CAS, PG (18:0/20:4) was moderately correlated with de novo lipogenesis (16:0/18:2n-6) (r = − 0.487, *p* = 0.044, Additional file 1: Fig. S2). There was no correlation between predictive metabolites and metabolic index. As was shown in Additional file 1: Fig. S3, de novo lipogenesis (16:0/18:2n-6) was found to have a negative correlation with pathological index, including steatosis, ballooning, NAS and SAF in MAFLD patients without CAS. For obese MAFLD without CAS, the correlation coefficients between de novo lipogenesis (16:0/18:2n-6) and l-cystine were 0.603 (*p* < 0.001) in the Pearson’s correlation test. SM (16:1/18:1) was positively correlated with de novo lipogenesis (16:0/18:2n-6) (r = − 0.474, *p* = 0.035) and TG (r = − 0.593, *p* = 0.006, Additional file 1: Fig. S4). There was no correlation between predictive metabolites and pathological index (Additional file 1: Fig. S5).

### Prediction of carotid atherosclerosis with a serum metabolite panel in nonobese and obese MAFLD patients

Based on the results of logistics analysis, ROC curve analysis was further conducted to evaluate the predictive value of metabolites for CAS. For nonobese MAFLD patients in deviation cohort, PE (14:1/24:1), PG (18:0/20:4), de novo lipogenesis (16:0/18:2n-6) and liver stiffness achieved significant area under the ROC curves (AUC, 0.67, *p* = 0.03; 0.63, *p* = 0.03; 0.79, *p* = 0.02; *p* = 0.75, *p* = 0.02, respectively). The combination of these four indexes resulted in a statistically higher AUC (0.92, *p* < 0.01, Fig. 3D). Likewise, in obese MAFLD patients of deviation cohort, age was found to be significant with an AUC of 0.84 (*p* < 0.001) followed by LFC (0.80, *p* < 0.001), de novo lipogenesis (16:0/18:2n-6) (0.73, *p* = 0.004), SM (16:1/18:1) (0.71, *p* = 0.004) and cystine (0.69, *p* = 0.015). The combination of these indexes attained a higher AUC of 0.91 (*p* < 0.001, Fig. 3D).

We further utilized the validation cohort to evaluate the value of the predictive models. In nonobese patients, PE (14:1/24:1), PG (18:0/20:4), de novo lipogenesis (16:0/18:2n-6) and liver stiffness exhibited significant AUCs (0.61, *p* = 0.03; 0.71, *p* = 0.013; 0.76, *p* = 0.02; 0.71, *p* = 0.004, respectively). Besides, the combination of these indexes attained an AUC of 0.94 (*p* < 0.001, Fig. 3D). With regard to obese patients, age resulted in an AUC of 0.81 (*p* < 0.001), followed by liver fat content (0.80, *p* < 0.001), de novo lipogenesis (16:0/18:2n-6) (0.76, *p* < 0.001), SM (16:1/18:1) (0.70, *p* = 0.007) and cystine (0.64, *p* = 0.02). The combination had a significant AUC of 0.89 (*p* < 0.001, Fig. 3D).

## Discussion

In this study, we revealed that metabolic profiles differed in nonobese and obese MAFLD patients with or without CAS. This is the first study to compare the metabonomic predictors of CAS among nonobese and obese MAFLD patients. Our study identified that the PG (18:0/20:4) was independently associated with CAS in nonobese MAFLD but not the obese ones, and the panel of PE (20:2/16:0), de novo lipogenesis (16:0/18:2n-6), PG (18:0/20:4) and liver stiffness were useful in the diagnosis of CAS in nonobese MAFLD patients with higher AUCs than the individual significant metabolites. Therefore, our study generated a novel non-invasive diagnostic modality for early CAS screening in nonobese MAFLD patients using metabolomics.

Several studies have indicated that alterations in phospholipid metabolism are related to the development of MAFLD and CVD. Circulating phospholipid patterns have been proven to be associated with metabolic risk factors, hepatic steatosis and inflammation severity in MAFLD [26, 27]. Some phospholipid classes, including lysophosphatidylcholines (lyso-PC C18:0, lyso-PC C17:0) and phosphatidylcholines (PCaa C36:3), have been shown a potential role in the pathogenesis of nonobese MAFLD. Such phospholipid species were also involved in CVD occurrence. Stegemann et al. had analysed 685 plasma samples of prospective study and revealed that levels of lysophosphatidylcholines (lyso-PC), cholesterol esters (CE), PC, PE, SM and triacylglycerols (TAGs) were associated with CVD and the strongest predictive metabolites were PE (16:1) and PE (36:5) over a 10-year observation [28]. Roe et al. further performed a secondary analysis of a cross-sectional study involving 296 older adults demonstrated that higher concentrations of PC were associated with a favourable cardiometabolic risk-factor profile (higher HDL-C, lower BMI, lower WC, and lower odds of hypertension and diabetes) [29]. Paapstel et al. conducted a study including 52 patients with coronary artery disease, 32 patients with peripheral arterial disease, and 40 healthy individuals revealed that significantly decreased serum levels of phosphatidylcholine (PC) and lysoPC species (e.g., PC aa C28:1, PC aa C30:0, PC aa C32:2, PC ae C30:0 and PC ae C34:2, lysoPC a C18:2) were observed in the patient groups compared with the healthy subjects [30]. PG was found to have positive genetic correlations (r: 0.64–0.82) with CVD from the Busselton Family Heart Study consisting of 4492 individuals from Western Australia [31]. However, phospholipid markers of MAFLD-associated CVD have not been reported yet. Our data found that a set of PC (18:2/20:2) and PE (20:2/16:0) were associated with CAS both in nonobese and obese MAFLD patients, while PG (18:0/20:4) was independently associated with CAS only in nonobese patients with MAFLD. PG is a glycerophospholipid that functions as a precursor for cardiolipin production, which participates in forming the mitochondrial inner membrane and maintaining mitochondrial transmembrane potential. Lower PG levels might reflect the defective CL remodelling. CAS may be explained by the disruption of the endothelial protective roles of CL in mitochondrial bioenergetics, autophagy/mitophagy, and mitogen-activated protein kinase (MAPK) pathway activation [32]. Thus our results verified the previous findings and suggested a potential role of PG as a marker for CVD in nonobese patients with MAFLD.

Increased de novo lipogenesis is a distinct characteristic of individuals with MAFLD [33]. Our results further revealed that increased de novo lipogenesis (the ratio of 16:0/18:2n-6 fatty acids) was a significant predictor of CAS in both nonobese and obese MAFLD patients, with AUCs of 0.75 and 0.64 respectively. It suggested that increased de novo lipogenesis might be an important cause of lipid metabolism disorder in both MAFLD and CVD. It has been further supported by a recent longitudinal prospective study conducted by Lai et al. which demonstrated that long-term levels of 16:0, 16:1n-7, and 18:1n-9 were each positively associated with incident total mortality and CVD-related mortality, whereas an inverse relationship existed with 18:0 fatty acids during a median follow-up of 13 years [34]. Furthermore, fatty acid synthase (FAS), the key de novo lipogenesis enzyme, has increased activity in the setting of diabetes and was proven to contribute to atheroprogression in carotid arteries of patients by changing lipid species levels [35]. Therefore, targeting liver lipid synthesis is of great significance for estimating the risks of progression to CVD in MAFLD.

Previous studies have shown that obesity and insulin resistance could lead to the increment of branched-chain amino acid (BCAAs) in serum. BCAAs have been regarded as the risk predictor of IR and CVD, and are correlated with the fat accumulation in the liver [36–38]. Zhang et al. hypothesized that in the liver, BCAAs activate mTOR, which inhibits autophagy and the FFA to triglycerides conversion, blocking the hepatic outflow pathway of FFAs and thus intensifying the lipotoxicity of FFAs. Furthermore, the blockade of autophagy increased cell death via apoptosis [39]. Elevation in BCAAs can result in the accumulation of catabolic intermediates, and incomplete oxidation of fatty acids and glucose contributing to mitochondrial dysfunction of pancreatic B-cells. BCAAs are essential to mediate the transport of carbon substrates for oxidation through the mitochondrial tricarboxylic acid cycle (TCA). Besides, an impaired upregulation of BCAA-mediated TCA is regarded as a significant contributor to mitochondrial dysfunction in liver steatosis. Acylcarnitine C3 and C5, generated by the catabolism of BCAAs in the liver and skeletal muscle were associated with the direct onset of obesity and IR [40]. Therefore, a high concentration of circulating BCAAs may account for the presence of lean MAFLD [39]. It has been found that dietary essential amino acids could ameliorate liver steatosis by inducing polyubiquitination of Plin2, a lipid droplet-stabilizing protein. Leucine and isoleucine, two BCAAs, were strongly associated with the activation of E3 ubiquitin ligase Ubr1, targeting Plin2 for degradation. It was demonstrated that the amino acid-induced Ubr1 activity was necessary to prevent steatosis in mouse livers and cultured human hepatocytes [41]. Furthermore, a previous cross-sectional study of 102 subjects indicated that elevated plasma glutamate levels were associated with increased CIMT, independently of established CVD risk factors [42]. Our study further demonstrated that cystine and l-leucine differentially expressed among six groups and cystine was associated with CAS only in the obese MAFLD patients.

Vitamins, including vitamin B, C, D and E, were found significantly associated with CAS and have been regarded as protective factors for CVD in the previous studies [43–46]. Besides, it is noteworthy that Vitamin E, an important antioxidant, was recommended by many guidelines as one of the medicines with potential therapeutic effects on liver steatosis [47, 48]. Up to now, no relevant evidence has been found about the association between vitamin deficiencies and CAS in nonobese MAFLD. However, our present study found that the serum level of α-tocopherol, the hydrolysate of vitamin E, had the revise relationship with CAS in nonobese MAFLD.

Although those pathways that are directly associated with CAS in nonobese MAFLD remain unclear, there is a distinct pathway correlated with inflammation in nonobese MAFLD; namely, dysfunctional visceral adipose tissue that leads to activating sterol regulatory element binding protein cleavage-activating protein (SCAP) nuclear factor kappa B (NFκB) pathway in macrophage inflammatory response, which induced increased fibrosis severity in the liver and which has been demonstrated as the independent progression markers of CAS [49–51].

There are several strengths in the present study. Firstly, we utilized HPLC-QTOF-MS and GC–MS to compare the entire metabolic profiles among nonobese and obese MAFLD patients with/without CAS. Secondly, a derivation cohort and a validation cohort were recruited to explore and test the performance of the predictive metabolites of CAS. The results indicated that the combination of several metabolites was considerably effective in predicting the presence of CAS. Thirdly, we utilized MRI-PDFF to quantify LFC and 2D-SWE to perform liver stiffness measurements. MRI-PDFF has been regarded as a novel non-invasive assessment of fat content in the whole liver and pancreas, while 2D-SWE has the highest diagnostic accuracy for staging fibrosis in MAFLD patients.

The main limitation of the present study is that we did not perform a long-term follow-up with the involved patients to validate whether these predictors can longitudinally detect changes in MAFLD patients with CAS. Additionally, the number of patients in the two cohorts was limited, although we confirmed that the number was sufficient to attain statistical significance. Also, we mainly focused on the comparison of metabolic characteristics via HPLC-QTOF-MS and GC–MS without performing metabolic pathway analysis or investigating gene polymorphisms that predispose those with MAFLD to the presence of CAS.

## Conclusions

Fifty-six metabolites belonging to amino acids, carbohydrates, vitamins, and lipid families were found to be significant in discriminating among nonobese and obese MAFLD patients with/without CAS and healthy individuals. The combination of PE (20:2/16:0), de novo lipogenesis (16:0/18:2n-6), PG (18:0/20:4) and liver stiffness were strong predictors of CAS in nonobese MAFLD patients. While for obese patients, the combination of cystine, SM (16:1/18:1), de novo lipogenesis (16:0/18:2n-6), age and LFC were correlated with CAS. Based on our findings, diagnostic models combining different metabolites according to BMI categories could improve the accuracy of identifying subclinical CAS, which highlights the necessity of establishing metabolic and individualized CAS screening among MAFLD patients with different BMIs.

## Supplementary Information

**Additional file 1: Figure S1.** Chromatogram of top 10 metabolites in the targeted metabolomics analysis. **Figure S2.** The correlation between the predictive metabolites of CAS [PE (20:2/16:0), PG (18:0/20:4) and de novo lipogenesis (16:0/18:2n-6)] and metabolic index among nonobese MAFLD. Group 1: nonobese MAFLD patients without CAS; Group 2: nonobese MAFLD patients with CAS. **Figure S3.** The correlation between the predictive metabolites of CAS [PE (20:2/16:0), PG (18:0/20:4) and de novo lipogenesis (16:0/18:2n-6)] and pathological index among nonobese MAFLD. Group 1: nonobese MAFLD patients without CAS; Group 2: nonobese MAFLD patients with CAS. **Figure S4.** The correlation between the predictive metabolites of CAS [l-cystine, SM (16:1/18:1) and de novo lipogenesis (16:0/18:2n-6)] and metabolic index among obese MAFLD. Group 3: obese MAFLD patients without CAS; Group 4: obese MAFLD patients with CAS. **Figure S5.** The correlation between the predictive metabolites of CAS [l-cystine, SM (16:1/18:1) and de novo lipogenesis (16:0/18:2n-6)] and pathological index among obese MAFLD. Group 3: obese MAFLD patients without CAS; Group 4: obese MAFLD patients with CAS.

## Data Availability

The datasets used and/or analyzed during the current study are available from the corresponding author on reasonable request.

## Abbreviations

MAFLD: Metabolic dysfunction-associated fatty liver disease
CVD: Cardiovascular disease
CIMT: Carotid intimal-medial thickness
CAS: Carotid atherosclerosis
BMI: Body mass index
WC: Waist circumstance
TG: Triglycerides
CHOL: Total cholesterol
FBG: Fasting blood glucose
ALT: Alanine aminotransferase
AST: Aspartate transaminase
UHPLC-QTOF-MS: Ultrahigh-performance liquid chromatography coupled with quadrupole time-of-flight mass spectrometry
GC-MS: Gas chromatography–mass spectrometry
LFC: Liver fat content
PFC: Pancreas fat content
MRI-PDFF: Magnetic resonance imaging-based proton density fat fraction
CAP: Controlled attenuation parameter
HOMA-IR: Homeostatic model assessment of insulin resistance
ASFT: Abdominal subcutaneous fat thickness
PLS-DA: Partial least squares-discriminant analysis
VIP: Variable importance in the projection
BCAAs: Branched-chain amino acid

## References

[1] Le MH, Yeo YH, Li X, Li J, Zou B, Wu Y (2021). 2019 global NAFLD prevalence: a systematic review and meta-analysis. Clin Gastroenterol Hepatol.

[2] Zhou F, Zhou J, Wang W, Zhang XJ, Ji YX, Zhang P (2019). Unexpected rapid increase in the burden of NAFLD in China From 2008 to 2018: a systematic review and meta-analysis. Hepatology.

[3] Lin Y, Gong X, Li X, Shao C, Wu T, Li M (2021). Distinct cause of death profiles of hospitalized non-alcoholic fatty liver disease: a 10 years' cross-sectional multicenter study in China. Front Med.

[4] Targher G, Byrne CD, Lonardo A, Zoppini G, Barbui C (2016). Non-alcoholic fatty liver disease and risk of incident cardiovascular disease: a meta-analysis. J Hepatol.

[5] Lee H, Lee YH, Kim SU, Chang KH (2021). Metabolic dysfunction-associated fatty liver disease and incident cardiovascular disease risk: a Nationwide Cohort Study. Clin Gastroenterol Hepatol.

[6] Mozaffarian D, Benjamin EJ, Go AS, Arnett DK, Blaha MJ, Writing Group Members (2016). Heart disease and stroke statistics-2016 update: a report from the American Heart Association. Circulation.

[7] Miptah HN, Ramli AS, Mohamad M, Hashim H, Tharek Z (2020). Non-alcoholic fatty liver disease (NAFLD) and the cardiovascular disease (CVD) risk categories in primary care: is there an association?. BMC Fam Pract.

[8] Shao C, Ye J, Li F, Lin Y, Wu T, Wang W (2020). Early predictors of cardiovascular disease risk in nonalcoholic fatty liver disease: nonobese versus obese patients. Dig Dis Sci.

[9] Sookoian S, Pirola CJ (2017). Systematic review with meta-analysis: risk factors for non-alcoholic fatty liver disease suggest a shared altered metabolic and cardiovascular profile between lean and obese patients. Aliment Pharmacol Ther.

[10] Sookoian S, Pirola CJ (2018). Systematic review with meta-analysis: the significance of histological disease severity in lean patients with nonalcoholic fatty liver disease. Aliment Pharmacol Ther.

[11] Deprince A, Haas JT, Staels B (2020). Dysregulated lipid metabolism links NAFLD to cardiovascular disease. Mol Metab.

[12] Qi S, Xu D, Li Q, Xie N, Xia J, Huo Q (2017). Metabonomics screening of serum identifies pyroglutamate as a diagnostic biomarker for nonalcoholic steatohepatitis. Clin Chim Acta.

[13] Chen Y, Li C, Liu L, Guo F, Li S, Huang L (2016). Serum metabonomics of NAFLD plus T2DM based on liquid chromatography-mass spectrometry. Clin Biochem.

[14] McGranaghan P, Saxena A, Rubens M, Radenkovic J, Bach D, Schleußner L (2020). Predictive value of metabolomic biomarkers for cardiovascular disease risk: a systematic review and meta-analysis. Biomarkers.

[15] Eslam M, Sarin SK, Wong VW, Fan JG, Kawaguchi T, Ahn SH (2020). The Asian Pacific Association for the Study of the Liver clinical practice guidelines for the diagnosis and management of metabolic associated fatty liver disease. Hepatol Int.

[16] Eslam M, Newsome PN, Sarin SK, Anstee QM, Targher G, Romero-Gomez M (2020). A new definition for metabolic dysfunction-associated fatty liver disease: an international expert consensus statement. J Hepatol.

[17] Gu Q, Cen L, Lai J, Zhang Z, Pan J, Zhao F (2020). A meta-analysis on the diagnostic performance of magnetic resonance imaging and transient elastography in nonalcoholic fatty liver disease. Eur J Clin Investig.

[18] Sung KC, Ryan MC, Wilson AM (2009). The severity of nonalcoholic fatty liver disease is associated with increased cardiovascular risk in a large cohort of nonobese Asian subjects. Atherosclerosis.

[19] Lee SH, Yun SJ, Kim DH, Jo HH, Park YS (2017). Severity of nonalcoholic fatty liver disease on sonography and risk of coronary heart disease. J Clin Ultrasound.

[20] Shao C, Ye J, Li F, Feng S, Wang W, Zhong B (2019). Different predictors of steatosis and fibrosis severity among lean, overweight and obese patients with nonalcoholic fatty liver disease. Dig Liver Dis.

[21] Dong Z, Luo Y, Zhang Z, Cai H, Li Y, Chan T (2014). MR quantification of total liver fat in patients with impaired glucose tolerance and healthy subjects. PLoS ONE.

[22] Ye J, Hu X, Wu T, Wu Y, Shao C, Li F (2019). Insulin resistance exhibits varied metabolic abnormalities in nonalcoholic fatty liver disease, chronic hepatitis B and the combination of the two: a cross-sectional study. Diabetol Metab Syndr.

[23] Gu Q, Cen L, Lai J, Zhang Z, Pan J, Zhao F (2021). A meta-analysis on the diagnostic performance of magnetic resonance imaging and transient elastography in nonalcoholic fatty liver disease. Eur J Clin Investig.

[24] Touboul PJ, Hennerici MG, Meairs S, Adams H, Amarenco P, Bornstein N (2012). Mannheim carotid intima-media thickness and plaque consensus (2004–2006–2011). An update on behalf of the advisory board of the 3rd, 4th and 5th watching the risk symposia, at the 13th, 15th and 20th European Stroke Conferences, Mannheim, Germany, 2004, Brussels, Belgium, 2006, and Hamburg, Germany, 2011. Cerebrovasc Dis..

[25] Wang C, Lv G, Zang D (2017). Risk factors of carotid plaque and carotid common artery intima-media thickening in a high-stroke-risk population. Brain Behav.

[26] Tiwari-Heckler S, Gan-Schreier H, Stremmel W, Chamulitrat W, Pathil A (2018). Circulating phospholipid patterns in NAFLD patients associated with a combination of metabolic risk factors. Nutrients.

[27] Hall Z, Bond NJ, Ashmore T, Sanders F, Ament Z, Wang X (2017). Lipid zonation and phospholipid remodeling in nonalcoholic fatty liver disease. Hepatology.

[28] Stegemann C, Pechlaner R, Willeit P, Langley SR, Mangino M, Mayr U (2014). Lipidomics profiling and risk of cardiovascular disease in the prospective population-based Bruneck study. Circulation.

[29] Roe AJ, Zhang S, Bhadelia RA, Johnson EJ, Lichtenstein AH, Rogers GT (2017). Choline and its metabolites are differently associated with cardiometabolic risk factors, history of cardiovascular disease, and MRI-documented cerebrovascular disease in older adults. Am J Clin Nutr.

[30] Paapstel K, Kals J, Eha J, Tootsi K, Ottas A, Piir A (2018). Inverse relations of serum phosphatidylcholines and lysophosphatidylcholines with vascular damage and heart rate in patients with atherosclerosis. Nutr Metab Cardiovasc Dis.

[31] Cadby G, Melton PE, McCarthy NS, Giles C, Mellett NA, Huynh K (2020). Heritability of 596 lipid species and genetic correlation with cardiovascular traits in the Busselton Family Heart Study. J Lipid Res.

[32] Shen Z, Ye C, McCain K, Greenberg ML (2015). The role of cardiolipin in cardiovascular health. Biomed Res Int.

[33] Lambert JE, Ramos-Roman MA, Browning JD, Parks EJ (2014). Increased de novo lipogenesis is a distinct characteristic of individuals with nonalcoholic fatty liver disease. Gastroenterology.

[34] Lai HTM, de Oliveira Otto MC, Lee Y, Wu JHY, Song X, King IB (2019). Serial plasma phospholipid fatty acids in the de novo lipogenesis pathway and total mortality, cause-specific mortality, and cardiovascular diseases in the cardiovascular health study. J Am Heart Assoc.

[35] De Silva GS, Desai K, Darwech M, Naim U, Jin X, Adak S (2019). Circulating serum fatty acid synthase is elevated in patients with diabetes and carotid artery stenosis and is LDL-associated. Atherosclerosis.

[36] Haufe S, Witt H, Engeli S, Kaminski J, Utz W, Fuhrmann JC (2016). Branched-chain and aromatic amino acids, insulin resistance and liver specific ectopic fat storage in overweight to obese subjects. Nutr Metab Cardiovasc Dis.

[37] Cheng S, Rhee EP, Larson MG, Lewis GD, McCabe EL, Shen D (2012). Metabolite profiling identifies pathways associated with metabolic risk in humans. Circulation.

[38] Lee CC, Watkins SM, Lorenzo C, Wagenknecht LE, Il'yasova D, Chen YD (2016). Branched-chain amino acids and insulin metabolism: the insulin resistance atherosclerosis study (IRAS). Diabetes Care.

[39] Zhang F, Zhao S, Yan W, Xia Y, Chen X, Wang W (2016). Branched chain amino acids cause liver injury in obese/diabetic mice by promoting adipocyte lipolysis and inhibiting hepatic autophagy. EBioMedicine.

[40] Bhupathiraju SN, Guasch-Ferré M, Gadgil MD, Newgard CB, Bain JR, Muehlbauer MJ (2018). Dietary patterns among Asian Indians living in the United States have distinct metabolomic profiles that are associated with cardiometabolic risk. J Nutr.

[41] Zhang Y, Lin S, Peng J, Liang X, Yang Q, Bai X (2022). Amelioration of hepatic steatosis by dietary essential amino acid-induced ubiquitination. Mol Cell.

[42] Lehn-Stefan A, Peter A, Machann J, Schick F, Randrianarisoa E, Heni M (2021). Elevated circulating Glutamate associates with subclinical atherosclerosis independently of established risk markers: a cross-sectional study. J Clin Endocrinol Metab.

[43] Chen FH, Liu T, Xu L, Zhang L, Zhou XB (2018). Association of serum vitamin D level and carotid atherosclerosis: a systematic review and meta-analysis. J Ultrasound Med.

[44] Woo KS, Kwok TC, Celermajer DS (2014). Vegan diet, subnormal vitamin B-12 status and cardiovascular health. Nutrients.

[45] Violi F, Nocella C, Loffredo L, Carnevale R, Pignatelli P (2022). Interventional study with vitamin E in cardiovascular disease and meta-analysis. Free Radic Biol Med.

[46] Moser MA, Chun OK (2016). Vitamin C and heart health: a review based on findings from epidemiologic studies. Int J Mol Sci.

[47] Cusi K, Isaacs S, Barb D, Basu R, Caprio S, Garvey WT (2022). American Association of Clinical Endocrinology Clinical Practice Guideline for the diagnosis and management of nonalcoholic fatty liver disease in primary care and endocrinology clinical settings: co-sponsored by the American Association for the Study of Liver Diseases (AASLD). Endocr Pract.

[48] Fan JG, Wei L, Zhuang H, National Workshop on Fatty Liver and Alcoholic Liver Disease, Chinese Society of Hepatology, Chinese Medical Association, Fatty Liver Disease Expert Committee, Chinese Medical Doctor Association (2019). Guidelines of prevention and treatment of nonalcoholic fatty liver disease (2018, China). J Dig Dis.

[49] Huang X, Yao Y, Hou X, Wei L, Rao Y, Su Y (2022). Macrophage SCAP contributes to metaflammation and lean NAFLD by activating STING-NF-κB signaling pathway. Cell Mol Gastroenterol Hepatol.

[50] DiStefano JK, Gerhard GS (2022). NAFLD in normal weight individuals. Diabetol Metab Syndr.

[51] Fracanzani AL, Petta S, Lombardi R, Pisano G, Russello M, Consonni D (2017). Liver and cardiovascular damage in patients with lean nonalcoholic fatty liver disease, and association with visceral obesity. Clin Gastroenterol Hepatol.

