# Supplementary material for: Metabolomics to identify fingerprints of carotid atherosclerosis in nonobese metabolic dysfunction-associated fatty liver disease
Source: J Transl Med. 2023 Jan 9;21:12. doi: 10.1186/s12967-022-03760-6 (PMC9830861; doi:10.1186/s12967-022-03760-6)
Supplement: Supplementary file 1 — Additional file 1: Figure S1. Chromatogram of top 10 metabolites in the targeted metabolomics analysis. Figure S2. The correlation between the predictive metabolites of CAS [PE (20:2/16:0), PG (18:0/20:4) and de novo lipogenesis (16:0/18:2n-6)] and metabolic index among nonobese MAFLD. Group 1: nonobese MAFLD patients without CAS; Group 2: nonobese MAFLD patients with CAS. Figure S3. The correlation between the predictive metabolites of CAS [PE (20:2/16:0), PG (18:0/20:4) and de novo lipogenesis (16:0/18:2n-6)] and pathological index among nonobese MAFLD. Group 1: nonobese MAFLD patients without CAS; Group 2: nonobese MAFLD patients with CAS. Figure S4. The correlation between the predictive metabolites of CAS [l-cystine, SM (16:1/18:1) and de novo lipogenesis (16:0/18:2n-6)] and metabolic index among obese MAFLD. Group 3: obese MAFLD patients without CAS; Group 4: obese MAFLD patients with CAS. Figure S5. The correlation between the predictive metabolites of CAS [l-cystine, SM (16:1/18:1) and de novo lipogenesis (16:0/18:2n-6)] and pathological index among obese MAFLD. Group 3: obese MAFLD patients without CAS; Group 4: obese MAFLD patients with CAS. [file 12967_2022_3760_MOESM1_ESM.docx]

**Additional file**

**Material and methods**

**Histological assessment**

Liver biopsy was performed in the right hepatic lobe under the ultrasound guidance using 18G Temno needles and avoiding major blood vessels and intrahepatic ducts. Two passes of liver specimens were attained for each patient to get the samples with at least 15 mm in length. The histological characteristics were evaluated and scored via the NASH clinical research network system. Discriminating normal control, mild, moderate and severe steatosis was defined by the presence of steatosis in <5% (S0), ≥5% (S1), ≥33.3% (S2) and ≥66.7% (S3) of hepatocytes according to histological analysis. Fibrosis was graded using the Kleiner Fibrosis Score (F0–F4). Two fixed pathologists with over 10 years of experience were blinded to all the clinical data and reviewed the liver specimens separately. There were 4 cases of scoring inconsistency, therefore, a third pathologist participated in the discussion to achieve a final consensus.

**Metabonomics**

All samples were maintained at‐80C until processed. For UHPLC-QTOF-MS, five hundred microliters of acetonitrile (ACN)-methanol (MeOH) (9:1, v/v) precooled to 4 °C was added to serum samples (100 μL each) in 2mL of Eppendorf tube and the mixture was extracted by ultrasound-assisted for 2 min (the ultrasound frequency and power were 40 kHz and 100 W, respectively). Then, the mixture was stored at -20 ℃ for 10 min to improve protein precipitation and centrifuged at 10 000 g for 15 min at 4 ℃. After that, the supernatant (100 μL) was collected and transferred to 2 mL sample vial and was analyzed by UHPLC-QTOF-MS.

Liquid chromatographic analysis was performed on a Waters ACQUITY UPLC Iclass system (Waters Ltd. USA) coupled with an ACQUITY UPLC BEH C18 column (2.1mm ×100 mm, 1.7 μm; Waters, Ireland). There were two mobile-phase used to gradient analysis. Mobile phase A was water and mobile phase B was isopropanol acetonitrile (1:1, v/v). Both mobile phases were modified with 0.1% formic acid for both positive and negative ion mode. The gradient was set as follows: 5-25% of B at 0-1 min; 25-60% of B at 1-6 min; 60-90% of B at 6-15 min; 90-95% of B at 15-17 min; 95-98% of B at 17-20 min; 98% of B at 20-22 min; 5% of B at 22.1-25 min. The column temperature was set to 38 °C and the injection volume was 1.0 μL. Flow rate was 0.4 mL/min. Fifteen injections of the QC sample were done to equilibrate the system initially prior to the injections of the 30 samples and run QC sample after every 3 injections of the samples.

Raw data were acquired using a SYNAPT G2-Si HDMS equipped with an electrospray ionization source (Waters Ltd., USA). Nitrogen gas was set as desolvation gas and cone gas. MS parameters were as follow both in positive and in negative ion mode: capillary voltage set at 2.5 kV, nebulizer gas at 6 bar, cone voltage at 35 kV, cone gas flow at 40 L/h, source temperature at 110 ℃, desolvation gas temperature at 350 ℃, desolvation gas flow at 700 L/h. The acquisition method parameters were as follow: mass scan range of 50 to 1,200 mass-to-charge ratio (m/z) was set for data acquisition in continuous acquisition mode both in MS mode and in MS^E^ mode. Scan time was set as 0.3 second per scan. MS^E^ high collision energy was 25 to 65 eV. For both ionization modes, a lock spray solution of 1 ng/μL leucine enkephalin in water/acetonitrile (1:1, V/V) contains 0.1 % formic acid at a flow rate of 5 μL/min was continuously acquired and used to correct data (m/z 556.2771 in positive mode, m/z 554.2615 in negative mode). The original data was imported into the Progenesis QI (Nonlinear Dynamics Waters, UK) software to extract the matched peak of filtering noise peak and EZinfo 3.0 for Waters (Umetrics, Sweden) software was utilized for statistical analysis.

Phospholipid compound identification and analysis based on the phospholipid cleavage law and online databases METLIN metabolite MSMS database (http://metlin.scripps. edu) and LipidMaps (http://www.lipidmaps.org). The identification results were further confirmed by accurate mass number and secondary ion fragments collected by high resolution mass spectrometry.

For GC-MS, add 1000 μl methanol (containing 10 μg/ml ribosol) to 100 μl serum, vortex for 1 minute, ultrasonic assisted extraction for 5 minutes, centrifuged for 10 minutes at 12000 rpm, retain the supernatant and spin dry to get the powder. Next, add 50μl of methoxy pyridine solution (15mg/ml) to the powder, vortex for 30s, place at 70 ℃ for 1 hour. Then, add 50μl of MSTFA (containing 1% TMCS), vortex for 1 minute, and react at room temperature for 30 minutes. Finally, add 100μl n-heptane, vortex for 30s, centrifuge at 1500 g for 5 minutes and take 100μl supernatant for GC−MS analysis.

An Agilent 7890 GC system equipped with a 5977-quadrupole mass selective detector (MSD, Agilent, Santa Clara, CA) and a HP-5MS column (40 m×0.25 mm inner diameter×0.25 μm film thickness, Agilent, Santa Clara, CA) was employed to acquire metabolic profiles of the derivatized products. A total of 1 μL of the sample was injected in split mode with an inlet temperature of 250°C. Helium was used as the carrier gas in constant flow mode (pressure 115 kPa; flow 1.3 ml/min). The initial oven temperature was set to hold at 60°C for 1 min, followed by a ramp to 325°C at 10°C/min, and finally held for 10 min. The mass spectrum was acquired in full-scan mode from m/z 50 to 550. Electron impact ionization at 70 V was operated with an ion source temperature of 230°C.The ionization was carried out in the electron-impact mode (70 eV) with the temperatures of the ion source and the transfer line were 230 and 250°C, respectively. The electron multiplier voltage was set automatically. Recorded mass spectra were compared with those stored in the National Institute of Standards and Technology (NIST) US Government library. Quantitative analysis was performed by measuring total ion current chromatographic peak areas.

Targeted metabonomics was performed on a Agilent 1290 Infinity II Liquid Chromatograph (Agilent Ltd. USA) coupled to an Agilent 6495A Triple Quadrupole Mass Spectrometer with Agilent C18 column (2.1*50mm 1.8um, Agilent Ltd. USA). There were two mobile-phase used to gradient analysis. Mobile phase A was water acetonitrile (60:40, v/v) and mobile phase B was water acetonitrile isopropanol (10:10:80, v/v). Both mobile phases were modified with 0.1% formic acid for both positive and negative ion mode. The gradient was set as follows: 60%-100% of B at 0-2min, 100% of B at 2-4.5min, 100% of B at 4.5-5min, 60% of B at 5-7min. The column temperature was set to 35 °C and the injection volume was 1.0 μL. Flow rate was 0.3 mL/min. Source conditions were positive mode electrospray ionization, drying gas temperature 275°C, drying gas flow 14 L/min, nebulizer pressure 40 psi, sheath gas temperature 400°C, sheath gas flow 11 L/min. Analytes were detected with multiple reaction monitoring (MRM) with one transition for quantification and one transition for qualification per compound.

**Figure S1**


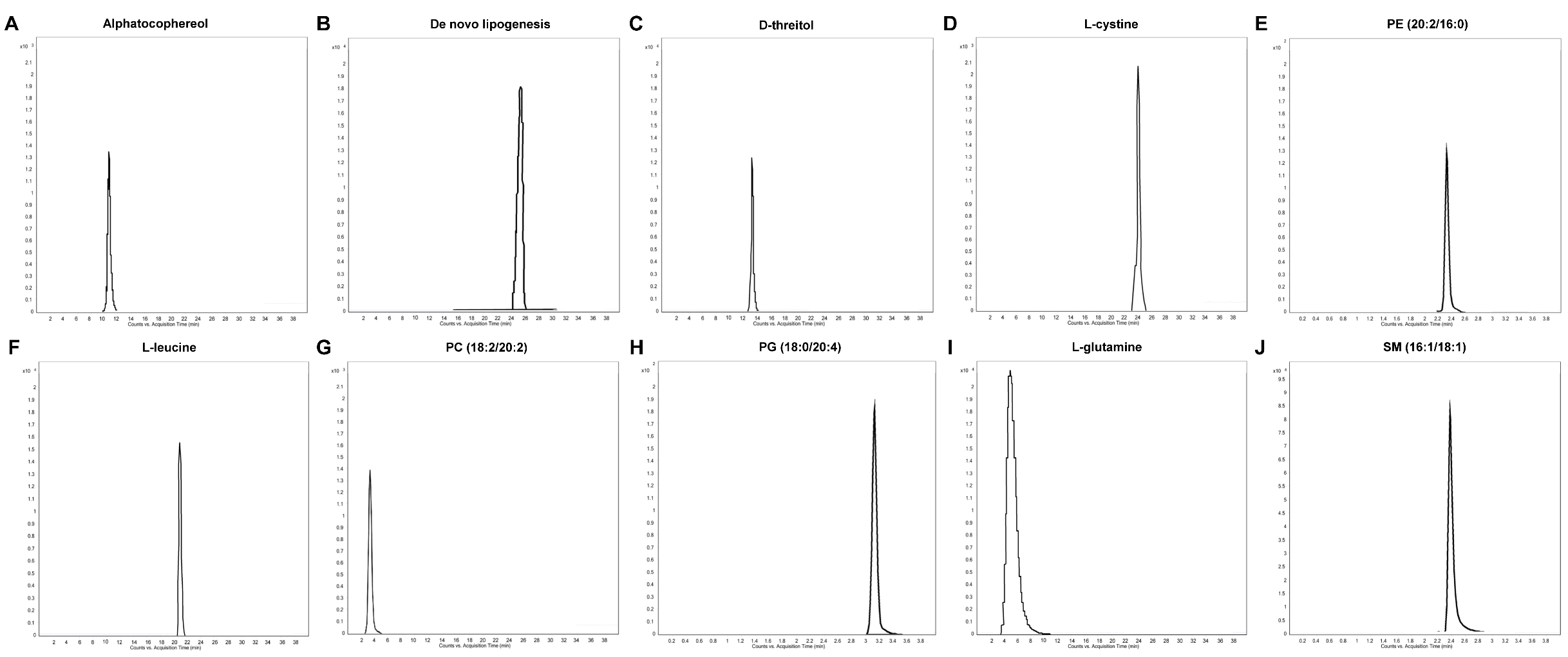


**
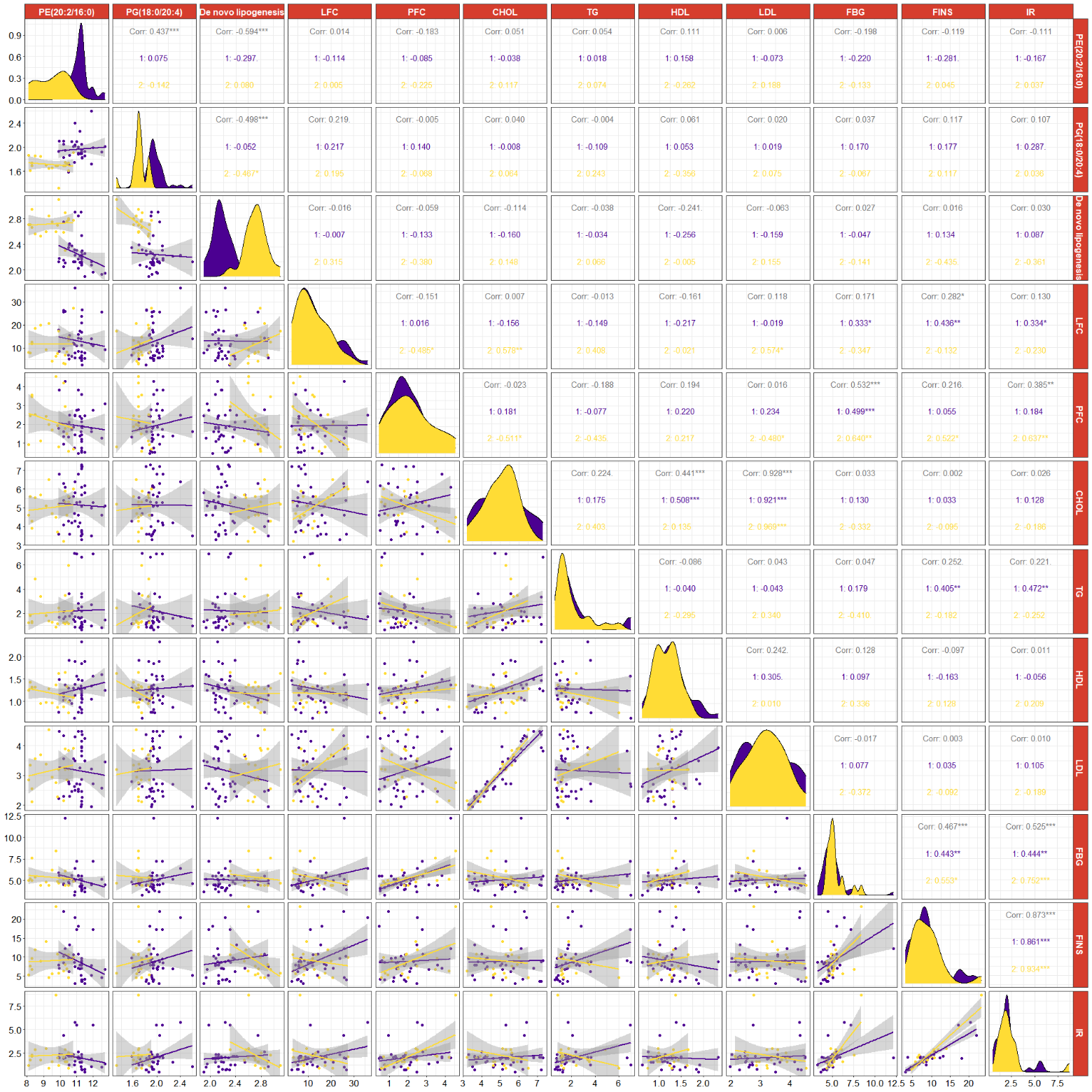
Figure S2**

**
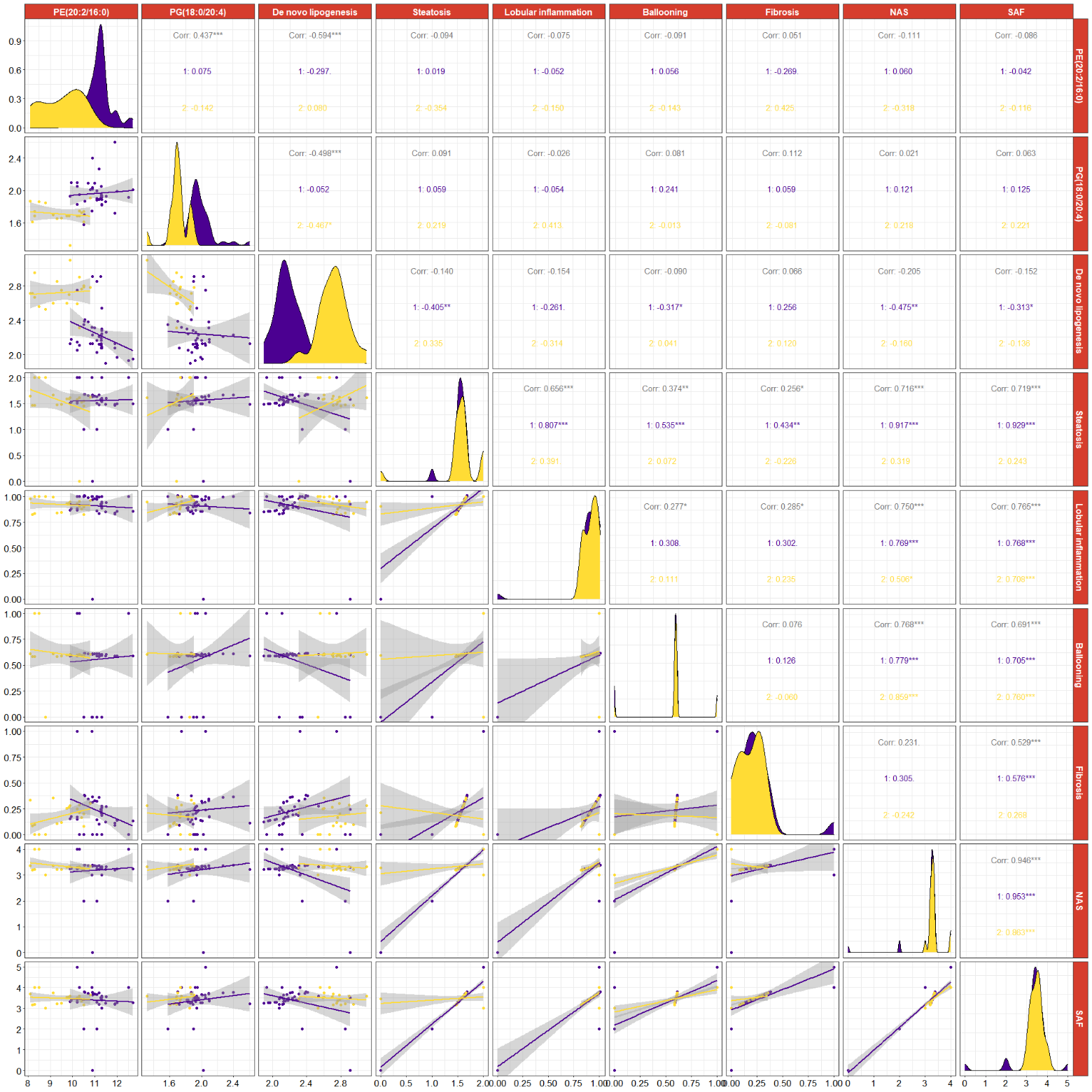
Figure S3**

**
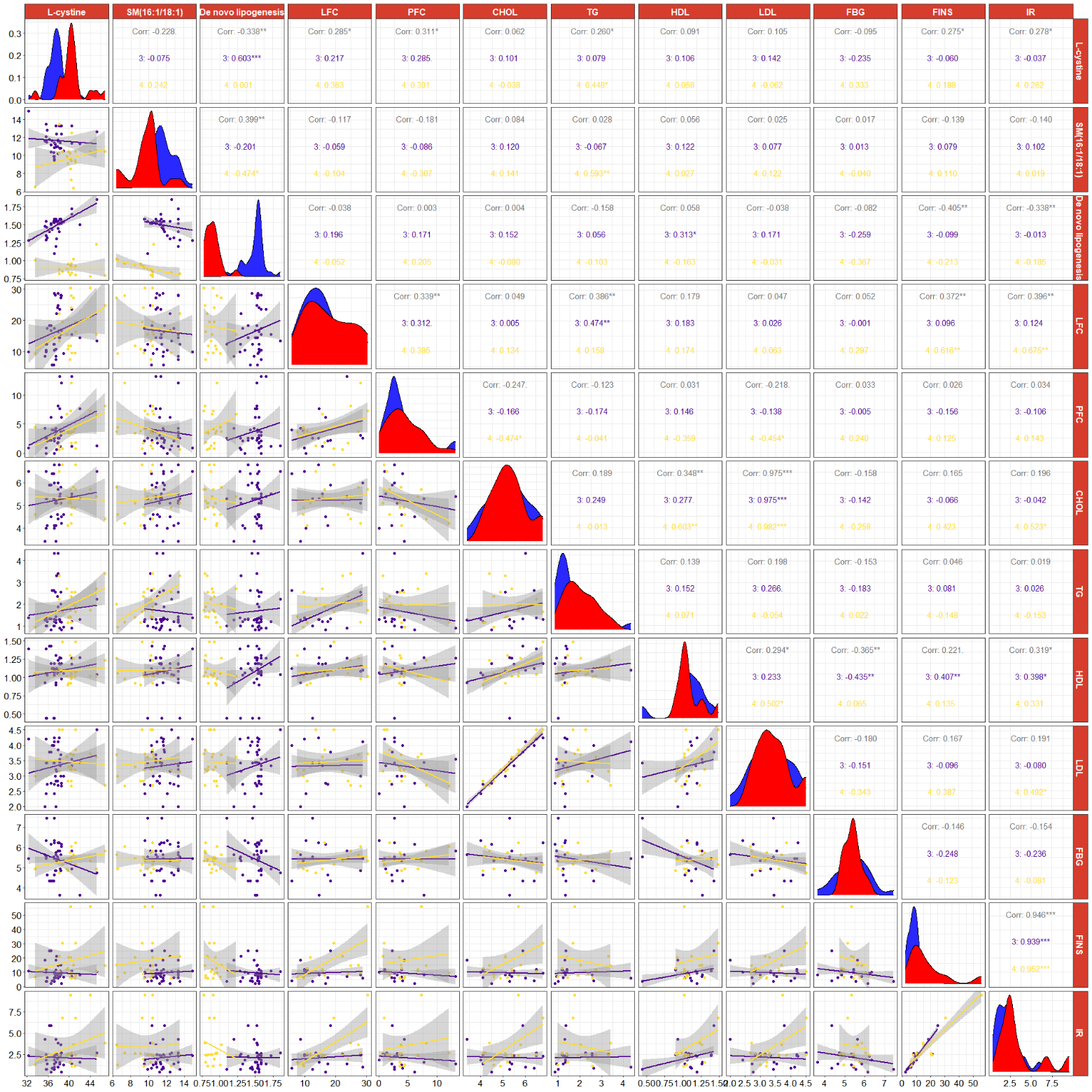
Figure S4**

**
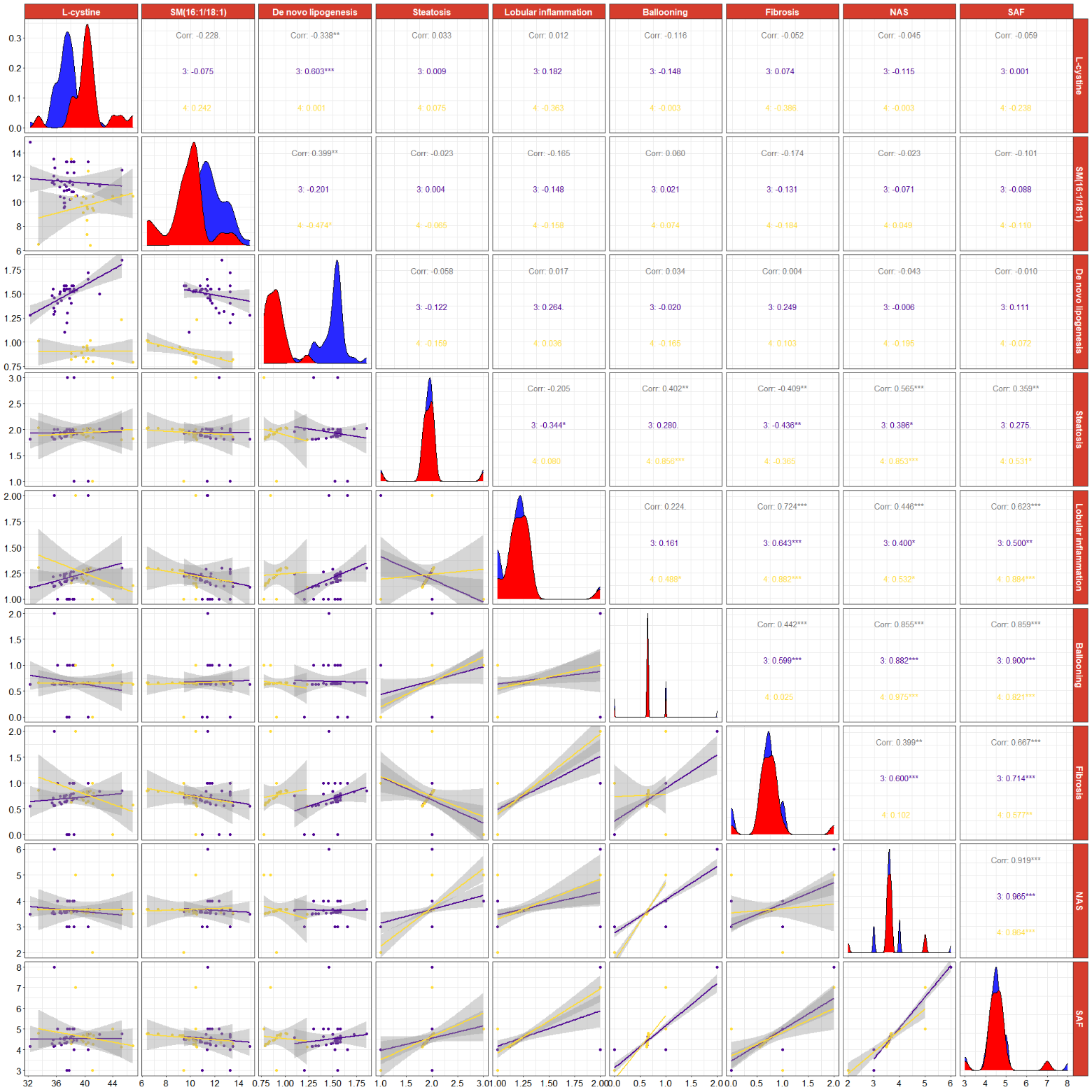
Figure S5**
